# Effect of Architected Structural Members on the Viscoelastic Response of 3D Printed Simple Cubic Lattice Structures

**DOI:** 10.3390/polym14030618

**Published:** 2022-02-05

**Authors:** Ahmed Abusabir, Muhammad A. Khan, Muhammad Asif, Kamran A. Khan

**Affiliations:** 1 School of Aerospace, Transport and Manufacturing, Cranfield University, Cranfield MK43 0AL, UK; Ahmed.Abusabir@cranfield.ac.uk; 2 Department of Mechanical Engineering, National University of Sciences and Technology, Karachi 75350, Pakistan; muhammadasif@pnec.nust.edu.pk; 3 Advanced Digital & Additive Manufacturing Center, Khalifa University of Science and Technology, Abu Dhabi P.O. Box 127788, United Arab Emirates; 4 Department of Aerospace Engineering, Khalifa University, Abu Dhabi P.O. Box 127788, United Arab Emirates

**Keywords:** 3D lattice structure, simple cubic lattice structures, plate-based lattice, shell-based lattice, truss-based lattice, ABS, PLA, 3D printing, FFF, viscoelastic behavior, relaxation, creep, cyclic loading

## Abstract

Three-dimensional printed polymeric lattice structures have recently gained interests in several engineering applications owing to their excellent properties such as low-density, energy absorption, strength-to-weight ratio, and damping performance. Three-dimensional (3D) lattice structure properties are governed by the topology of the microstructure and the base material that can be tailored to meet the application requirement. In this study, the effect of architected structural member geometry and base material on the viscoelastic response of 3D printed lattice structure has been investigated. The simple cubic lattice structures based on plate-, truss-, and shell-type structural members were used to describe the topology of the cellular solid. The proposed lattice structures were fabricated with two materials, i.e., PLA and ABS using the material extrusion (MEX) process. The quasi-static compression response of lattice structures was investigated, and mechanical properties were obtained. Then, the creep, relaxation and cyclic viscoelastic response of the lattice structure were characterized. Both material and topologies were observed to affect the mechanical properties and time-dependent behavior of lattice structure. Plate-based lattices were found to possess highest stiffness, while the highest viscoelastic behavior belongs to shell-based lattices. Among the studied lattice structures, we found that the plate-lattice is the best candidate to use as a creep-resistant LS and shell-based lattice is ideal for damping applications under quasi-static loading conditions. The proposed analysis approach is a step forward toward understanding the viscoelastic tolerance design of lattice structures.

## 1. Introduction

A new generation of engineering materials, known as lattice structures (LSs), has recently found applications in biomedical [1], aerospace [2] and automotive [3]. Notable properties of LSs include their low density and high specific thermal, electrical and mechanical properties, energy absorption, and ability to reduce noise/vibration [4,5,6]. The overall response of LSs depend on the relative density, solid base material, and topology of the microstructure. For damping and energy absorption applications, a better understanding of the relationship between microstructure of the LS and their effective viscoelastic properties is required to obtain desired performance [7,8].

LSs consist of a solid skeleton and air pores. The architecture of microstructure influences their mechanical behaviors. Numerous architectures were proposed in the literature to describe the microstructure of LS. The architected LSs are classified into two categories: open-cell and closed-cell foams, with either a random or periodic arrangement [9]. Earlier design of three dimensional (3D) networks of LSs are usually designed using discrete structural members such as struts or truss members. The microstructure, such as, rhombic dodecahedron [10], tetrakaidecahedron [11,12], cubic [6,13,14], Kelvin [15], Gibson-Ashby [16] and gyroids [17] have been studied. Analytical solutions for the effective response of these LSs were obtained through beam theory for elastic behavior [12,18,19,20] and viscoelastic behavior [21,22,23]. For more complicated architected LS, finite element homogenization method has been used to predict the elastic [24,25,26,27,28,29] and viscoelastic [22,30] responses.

Recently, three-dimensional network structures have been developed with interesting geometries derived from atomic crystal structures system [31]. The network of these lattice structures can be constructed with different structural members such as truss-, plate-, or shell-based (triply periodic minimal-surface (TPMS)) [32,33]. Out of these structural members, the plate-based lattice structures [34,35], offer superior stiffness which makes them excellent candidates for load-bearing applications. However, the shell based LSs such as TPMS demonstrated good energy absorption characteristics. Tancogne-Dejean et al. [36] showed that the specific energy absorption of plate-based LSs is around 45% greater than that of truss-based LSs. The elastic and viscoelastic properties of these lattice structures have been studied and investigated using the finite element method (FEM). Khan et al. used micromechanical homogenization approach to compute the apparent viscoelastic behavior such as creep, relaxation under quasi-static loading and dynamic behavior under cyclic excitation [37,38], and [39]. Previous studies highlighted the excellent viscoelastic response of the architected LSs [40]. Comprehensive studies have been conducted using theoretical and simulation approaches to investigate the properties of cellular solids; however, very limited experimental investigations have been undertaken to determine the viscoelastic response of polymeric LSs [15,40,41]. Moreover, the effect of architected structural member and base material on the viscoelastic response of 3D printed lattice structure has not been investigated

The revolution and growth in additive manufacturing have allowed the fabrication of complex and precise geometries of LSs. Additive manufacturing (AM) offers high flexibility of design and rapid prototyping. In the recent review article, it has been discussed that AM can reduce the production cost of complex components and can be implemented not only for prototyping but also production using different approaches in design [41]. Additive Manufacturing technology has enabled the porosity and architecture of cellular solids to be controlled; therefore, the density and mechanical properties can be tailored [42] for several applications [43]. Additive manufacturing includes several processes; however, the 3D printing technology using material extrusion (MEX) process [44] has been widely used to fabricate complex geometries such as cellular solids. Moreover, the base materials have significant influenced on the design of LSs. The LSs should be able to contribute to the functional purpose of structure with excellent damping performance, strength-to-weight ratio, and others. Thermoplastic polymers have been widely utilized in the fabrication of cellular solids due to their adaptability for 3D printing and their unique properties. The most utilized polymers are acrylonitrile butadiene styrene (ABS), and polylactic acid (PLA) [7,8]. The comparison of the flexural properties of ABS, PLA and a PLA–wood composite manufactured through MEX process has been presented [45]. Several authors have extensively studied the manufacturing of PLA using MEX process such an in-process monitoring of temperature evolution, multiscale damage and fatigue modeling of PLA [46,47,48]. The influence of process parameters has also been investigated on the mechanical properties [49], impact resistance properties [50] and interlayer adhesion on the tensile strength of 3D printed PLA [51].

In this study, the effect of architected structural member geometry and base material on the viscoelastic response of 3D printed lattice structure has been experimentally investigated. The LSs possessing simple cubic symmetry based on plate-, truss-, and shell-type structural members were considered to describe the microstructure of the LSs. The proposed LSs were fabricated with two materials, i.e., PLA and ABS using the material extrusion (MEX) process. The quasi-static compression response of lattice structures was investigated, and mechanical properties were obtained. Then, the creep, relaxation and cyclic viscoelastic response of the lattice structure were characterized and some interesting conclusions were presented.

## 2. Methodology

### 2.1. Design of Lattice Architecture and Manufacturing

In this study, the three lattice microstructures of simple cubic family were considered. The three designs are named as simple cubic truss-based lattice (SCTL), simple cubic plate-based lattice (SCPL), and simple cubic shell-based lattice (SCSL). The SCTL, SCPL, and SCSL unit cells consist of struts, plate and shell, respectively. The arrangement of these structural members yield simple cubic LSs. Solidworks software was used to model the considered designs. The 3D designs were made with overall dimensions of 25 × 25 × 25 mm^3^. The investigation was conducted using two polymeric materials: Polylactic acid (PLA), and acrylonitrile butadiene styrene (ABS). Raw materials of ABS and PLA were procured in the form of filament with 1.75 mm thickness. The specifications of the utilized materials are shown below in Table 1 as provided by the manufacturing company.

Additive manufacturing based on material extrusion (MEX) process, was adopted to fabricate all specimens. In this study, we employed the Raised3D Pro2 printer, which is equipped with a 0.4 nozzle. Several attempts were made to attain the best designs in terms of lightweight, manufacturability, and flexibility. The printing parameters that were given using software Idea Maker are shown below in Table 2.

For all specimens, the faces of the infill were perpendicular to the direction of the build (out-of-plane). All samples were printed with a raft platform to ensure the flatness of the base and stability throughout the printing process. Concerning solid infill density, all candidates were designed with 27% solid infill density. Table 3 shows the unit cell CAD design, the LS with array of 5 × 5 × 5 unit cells, the design and printing parameter, and the fabricated LSs made of PLA and ABS. Throughout this study, the investigated samples will be referenced by the assigned ID codes shown in Table 4.

### 2.2. Experiments

**Figure 1:**
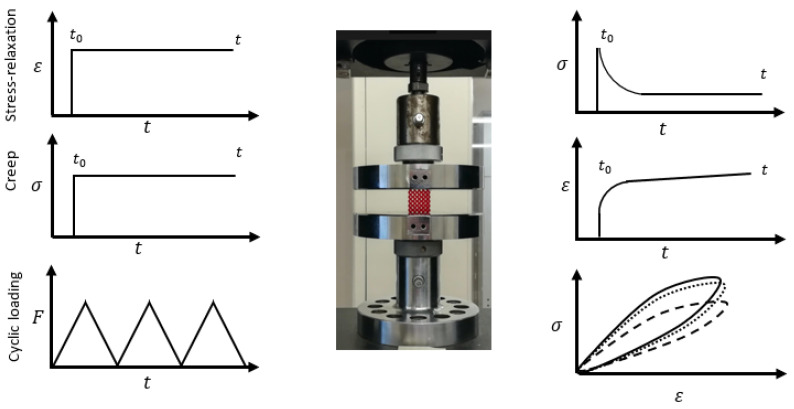
Experiment set-up and loading program.

Four experiments were performed to understand the mechanical properties and time-dependent behavior of the 3D printed polymeric LSs, i.e., quasi-static compression test, stress relaxation test, creep test, and compressive cyclic loading test, as shown in Figure 1. The experiments were conducted using an Instron universal testing machine with 5KN and 30KN load cells. The crosshead speed was 2.5 mm/min in all tests, chosen based on ASTM D1621-16 [52]. A pre-load was applied to guarantee a full initial contact between plates and specimen; all tests were conducted at room temperature. The experimental setup is shown in Figure 1. Pre-experimenting, the relative density of considered specimens were measured using a weight scale and Equation (1)

(1) $$\bar{\rho }=\frac{\rho_{c}}{\rho }$$

where $\bar{\rho }$: relative density, $\rho_{c}$: density of cellular solid, ρ: density of solid material.

#### 2.2.1. Quasi-Static Compression Test

First, the quasi-static compression test was performed until fracture. The quasi-static compression test was performed according to ASTM D1621-16 “Standard Test Method for Compressive Properties of Rigid Cellular Plastics”. The specimens were placed between the compression plates ensuring that the specimen centerline was aligned with the load cell centerline. Pre-loading was applied to ensure the stability of the samples and full initial contact between plates and specimens. The LSs were compressed at a constant crosshead speed of 2.5 mm/min and the effective stress–strain behavior was recorded. Many interesting characteristics of LS such as elastic modulus (*E*) and specific stiffness (*C*) were calculated using Equations (2) and (3).

(2) $$E=\frac{\sigma }{\varepsilon }$$

(3) $$C=\frac{E}{\bar{\rho }}$$

#### 2.2.2. Stress Relaxation Test

A stress relaxation test is necessary to understand the viscoelasticity behavior (time-dependent response), in which the specimen is compressed and held at a certain displacement; accordingly, the stress relaxation response is recorded as a function of time. The relaxation response can be measured by calculation stress-relaxation modulus using Equation (4).

(4) $$E_{sr}=\frac{\sigma_{t}}{\varepsilon_{0}}$$

The samples made from the different materials were compressed to the same strain level called effective strain. The effective strain should be on or below the yield point, which was determined using the data obtained from the quasi-static compression test. It was considered to be a value below the least yield limit among the three samples. Table 5 shows the effective strain levels used during stress relaxation test. The stress relaxation test were performed according to ASTM E328 − 21: Standard Test Methods for Stress Relaxation for Materials and Structures [53]. In this study, the displacement was applied on the specimen at the strain rate of 2.5 mm/min until reaching the desired displacement. The position (displacement) was held constant for 30 min and the stress relaxation response was recorded as a function of time.

#### 2.2.3. Creep Test

Viscoelastic behavior can also be measured by creep testing, in which constant stress is applied for a period of time and changes in strain are observed as a function of time. The viscoelastic behavior can be measured by finding creep compliance (J) using Equation (5). The creep test was performed according to ASTM D2990 − 17: Standard Test Methods for Tensile, Compressive, and Flexural Creep and Creep-Rupture of Plastics [54]. Table 6 shows the forces levels used during creep test. Here, the sample was compressed with a strain rate of 2.5 mm/min to the predetermined load limit and held constant for 30 min. While constant stress was applied, the strain will continue to increase with time and therefore recorded.

(5) $$\;J_{t}=\frac{\varepsilon_{t}}{\sigma_{0}}$$

#### 2.2.4. Compressive Cyclic Loading Test

The viscoelastic phenomenon and energy dissipation behavior of cellular materials can be observed by loading and unloading a specimen at a constant strain rate. The compressive cyclic loading test involves an appropriate repeating pattern of loading-unloading. The test may be conducted with a peak strain-controlled, or peak stress-controlled technique. In this study, the experiments were carried out with a peak stress-controlled method and the specimens were compressed with a strain rate of 2.5 mm/min to the predetermined load limit. The testing parameters are illustrated in Table 7. In total, three loading-unloading cycles were applied, and the load-displacement hysteresis loop were recorded. OriginLab software was used to calculate the area under the hysteresis curve, which represents the amount of energy absorption.

## 3. Results and Discussion

In this section, the data obtained from the experiments described above will be shown, analyzed, and discussed. A weight scale was used to measure the weight of the 3D printed specimens, then the relative density was calculated using Equation 1 as shown in Table 8.

The measured values show that all ABS samples having almost the same weight with a variation of ±0.06 (1.5%), similarly shown in all PLA specimens with a variation of ±0.16 (3%). The equality in weights verifies that the initial designs have the same solids infill density and the excellent accuracy of the manufacturing process. Several factors may have contributed to the slight variations, such as the uncertainty of the scaling device, the surrounding conditions in the lab, or minor uncertainties in the design or fabrication process.

### 3.1. Quasi-Static Compression Test

**Figure 2:**
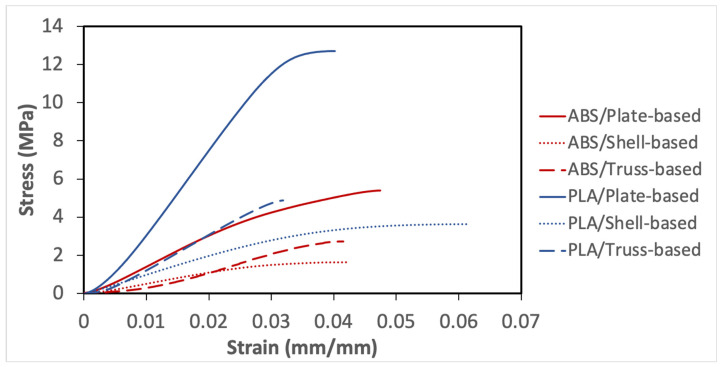
Compression stress–strain curves for the investigated samples.

Figure 2 shows the compression stress–strain curves for the investigated LSs. The stress–strain curve provides the mechanical behavior of LSs and could help to find the Young’s modulus and yield strength. The main purpose of this test is to obtain the linear stress–strain limit so that the effective load, and strain levels can be identified for creep, stress relaxation, and cyclic loading-unloading tests. It can be observed that the overall compressive behavior of LSs depends mainly on its microstructural design and relative density, and the mechanical properties of the base material. Generally, the higher the density, the higher the collapse stress. As defined early, PLA has a higher density than ABS, 1.25 and 1.03 g/cm^3^, respectively. Therefore, the fracture stress of the PLA samples is higher than that of the ABS specimens, as illustrated in Figure 2. With regards to the effect of the architected structural member geometry, it is evident that plate-based lattices are stiffer than others, followed by truss-based lattices then shell-based lattices made of the same material and relative density.

The Young’s modulus values were determined through the tangent value of the initial slope of the stress–strain curves, by using Equation 2 and the values Young’s Modulus are shown in Table 9. The plate-based lattice in both materials has the highest Young’s modulus values, and the least value of Young’s modulus belongs to the shell-based lattice. Moreover, another interesting property that can be obtained from the stress–strain curve is the specific stiffness, whereby the stiffness-to-density ratio can be measured using Equation 3; specific stiffness values are shown in Table 9.

Another important point to be noticed that the yield limit was not clearly defined as the LSs demonstrated nonlinear stress–strain response. The method of offset point was used to compute the yield point, that indicates the limit of elastic behavior and the beginning of plastic deformation. Table 9 shows the yield stress for the considered samples, which was important to be identified for subsequent experiments.

**Figure 3:**
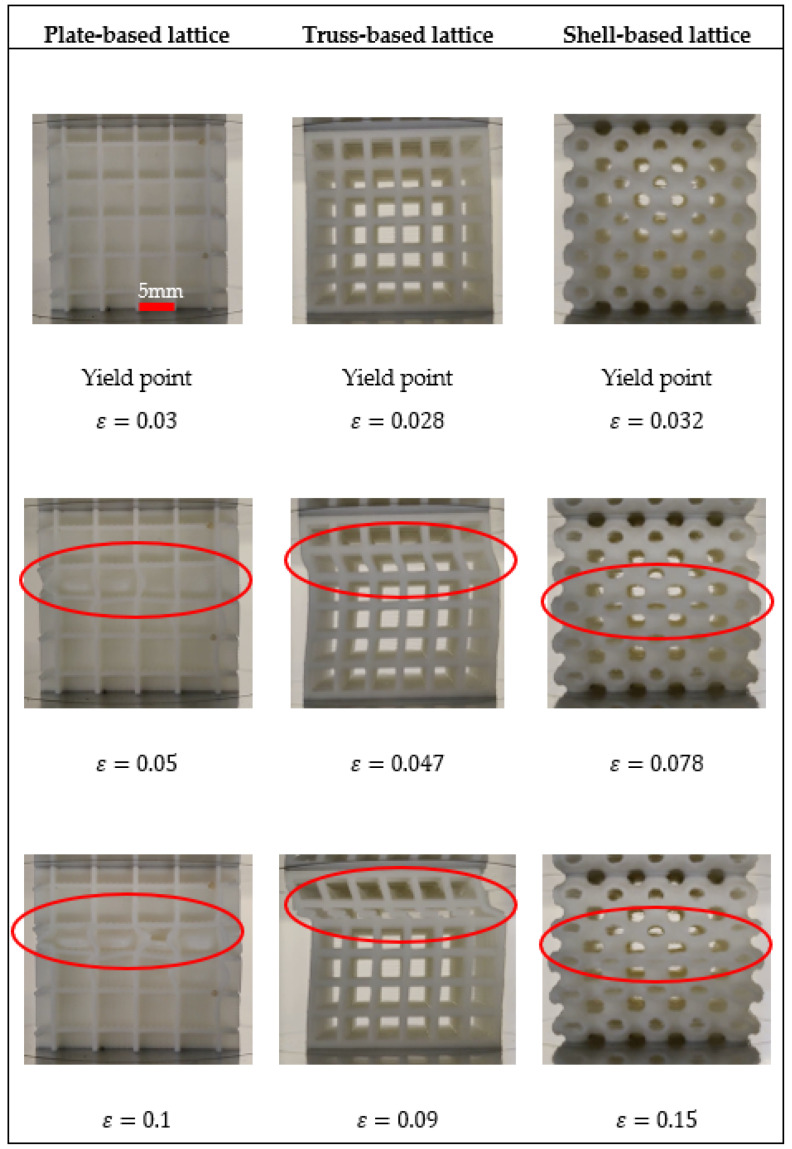
Deformation mechanism of the samples under investigation.

We investigated the architected structural member geometry on the deformation mechanism. All the three structures were deformed under uni-axial compression and representative pictures were taken during the tests at different strain levels as presented in Figure 3. Noticeably, there is no physical failure in the identified yield point as shown in the 1st row in Figure 3. Moreover, it was observed that buckling occurred when compressive strain reached to some critical value and consequently led to rapid and dramatic changes of the material microstructure, as illustrated in the 2nd row in Figure 3 (in which all three structures demonstrated clear buckling). Subsequently, the middle region of structural members reached to a completely collapsed and then the deformation progressed to the neighboring cells. The plate-based lattice deformation occurred by compressing layers over each other, while truss-based lattice deformed due to buckling of its struts, whereas shell-based lattice deformed by squeezing its unit cells.

Generally, it was observed that all samples have deformed in a stretching-dominated manner; however, each specimen has its characteristics. For examples, the high stiffness in plate-based lattice is due to its plates involvement to carry load capacity and the integration or configuration of the plate-based structure. On the other hand, when a truss-based lattice experiences a compression load, and most of the load is carried by struts located in the longitudinal direction of the force, which means more stress concentration in thin struts. Therefore, vertical struts are the first to fail via buckling. Moreover, shell-based lattice has a novel geometry that doesn’t contain struts or walls, the advantages of its architecture were observed during the experiment, whereby it exhibited great extension, resulting from the uniform distribution of the stresses.

### 3.2. Stress Relaxation Test

**Figure 4:**
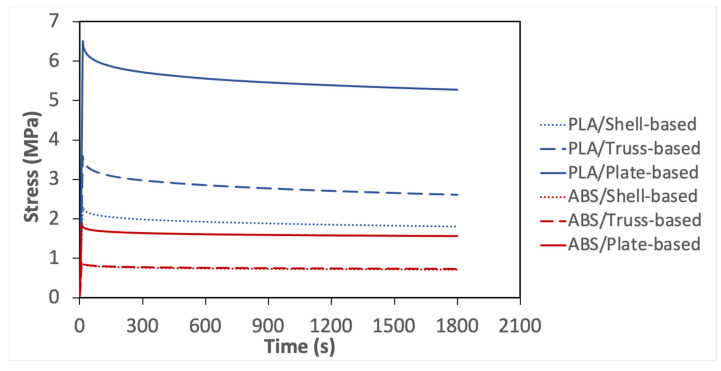
Stress relaxation response of all considered specimens.

The stress relaxation experiment was undertaken according to the procedure explained in above methodology section. Equal effective strain was applied in each sample made of the same material, based on the outcomes of quasi-static compression test, the elastic limit of PLA samples is higher than ABS samples. Therefore, PLA samples experienced higher initial stress than ABS, as shown in Figure 4.

As can be seen in Figure 4, the stress relaxation curves can be divided into three stages. The first stage is the effective elastic stage, in which the specimens were compressed to the predetermined displacement and then held for 30 min. This initial displacement determined the starting point of stress relaxation. Then, the stress relaxation started after the first stage and can also be divided into two stages: transient stage and stable stage, representing the regions of decreasing stress relaxation rate and near-constant stress relaxation rate, respectively.

Figure 4 shows the plate-based lattice experienced the greatest stress to deform to the predetermined strain level, followed by the truss-based lattice. In contrast, the shell-based lattice demonstrated the least load bearing capacity. These results are due to the stiff plate-based structure, which is aligned with the conclusions drawn from the quasi-static test. As shown in Figure 4, all considered samples exhibited different stress relaxation behavior over time, which demonstrates that different viscoelastic mechanism exists in each specimen.

For further analysis, the percentage of the normalized stress was calculated and shown in Table 10. It was found that the shell-based lattice outperformed the truss-based lattice and the plate-based lattice in terms of normalized stress over time. In addition, to determine the viscoelastic response from the stress relaxation test, the stress relaxation moduli were calculated using Equation 4. Then, the stress-relaxation moduli were converted to the relative moduli to compare based on the two considered materials as listed in Table 10. From the calculated values, it can be seen that the shell-based lattices have the greatest viscoelastic behavior, followed by the truss-based lattice, then the plate-based lattice. The outperformance of the shell-based lattice is due to its smooth geometry and curvature interconnections, by which the stress concentration is reduced, and the applied stress distributed uniformly. However, the stiffness of plate-based lattice has an adverse effect on the viscoelastic response. From the relative modulus values, it can be concluded that ABS samples have better viscoelasticity than that of PLA, resulting from the less stiffness and better elongation of ABS.

### 3.3. Creep Test

**Figure 5:**
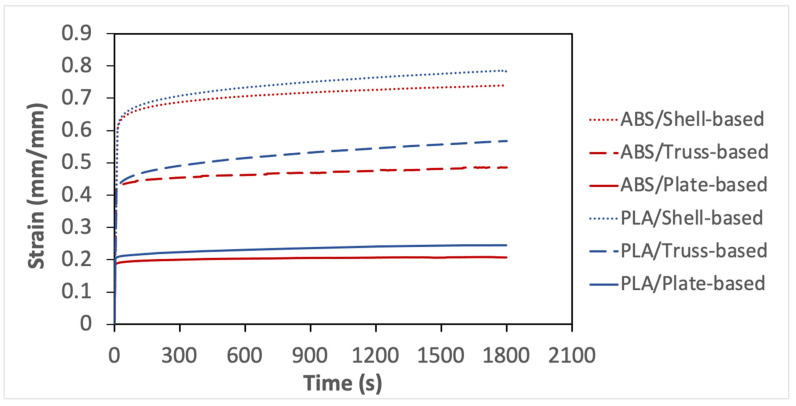
Strain–time plots of creep test for all considered specimens.

The creep experiment was conducted following the procedure discussed earlier in the methodology section. The data obtained from the creep test are plotted in Figure 5. The shell-based lattice experienced the highest initial strain level, while the least value of applied strain belongs to the plate-based lattice. Those results are because all samples made of the same material have compressed to the same effective stress level and conform to the conclusions of previous experiments. The shell-based lattice was the compliant, while plate-based lattice was the stiffest.

Additionally, the creep curves can be divided into three stages: the first stage is the elastic deformation stage, in which a uniaxial compression load was applied at a constant rate to the specimen until it reached the predetermined stress level and then be held. In this stage, the slope of PLA specimens is higher than that of ABS specimens due to the higher stiffness of PLA, which required more strain energy. The creep started after the first stage and can be divided into two stages: the transient stage and near-stable stage. All samples demonstrated creep deformation over time, which verifies the nature of viscoelastic behavior. However, only plate-based LSs demonstrated steady state creep strain for the considered testing time. The percentage of the creep strain increase was calculated and shown in Table 11. All shell-based lattice outperformed the truss-based lattice and plate-based lattice in terms of creep response. Moreover, the creep compliance was calculated using Equation 5. whereby the greatest compliance behavior belongs to the shell-based lattices, followed by the truss-based lattice, then the plate-based lattice. The is again because of the smooth interconnection of the shell-based lattice and uniform stress distribution and transfer from one cell layer to another. It is concluded that the viscoelastic behavior of ABS is better than that of PLA due to the softness and elongation of ABS.

### 3.4. Compressive Cyclic Loading Test

**Figure 6:**
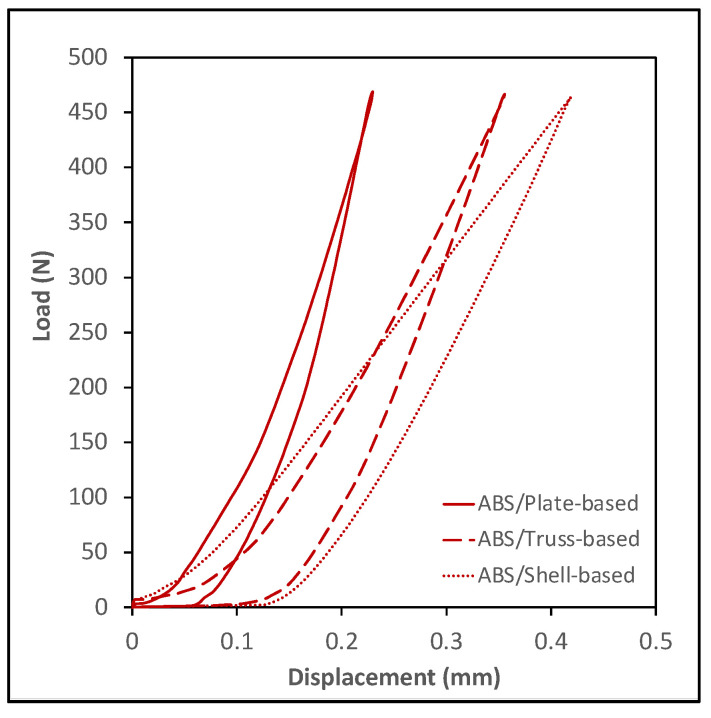
Cyclic loading of ABS specimens.

**Figure 7:**
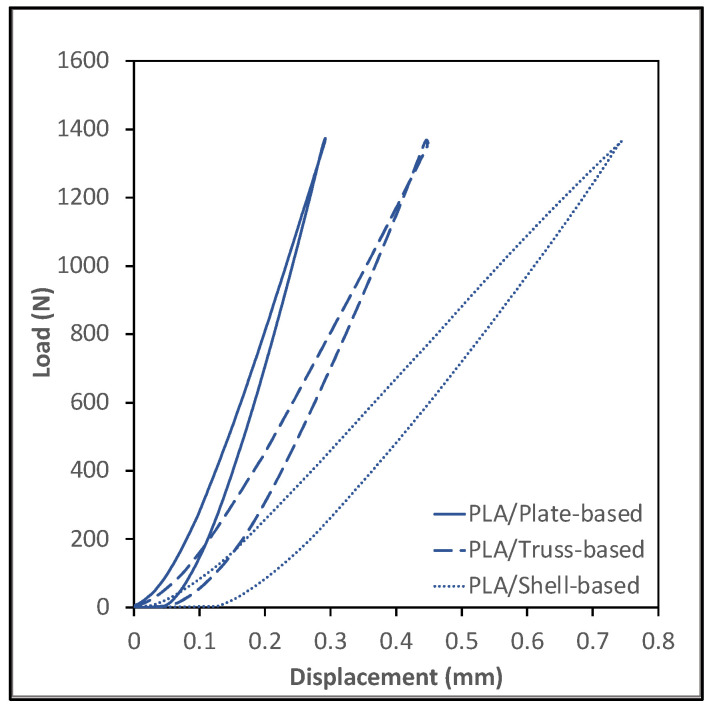
Cyclic loading of PLA specimens.

**Figure 8:**
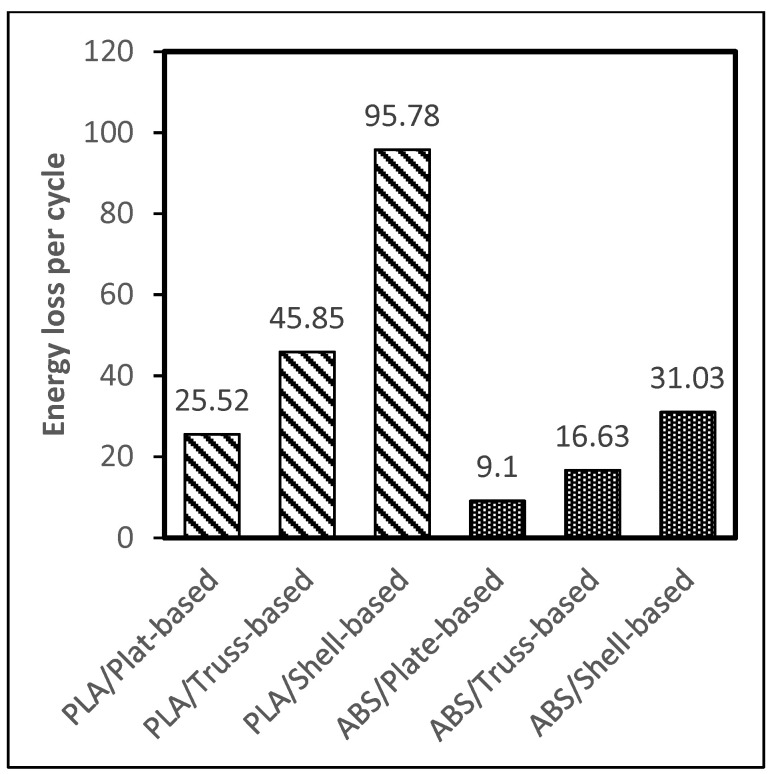
Area of hysteresis curves of the considered specimens.

The compressive cyclic loading experiment was conducted following the procedure described in the methodology section. Figure 6 and Figure 7 show plots of the load vs. displacement values for ABS and PLA samples. All tested specimens demonstrated a viscoelastic behavior and formed a hysteresis loop. The shape of the hysteresis curves dictates the energy dissipation capacity of LSs. The samples can be ranked by estimating the area inside the hysteresis loop; the wider loop means the better damping performance, energy dissipation capacity, or viscoelastic behavior. Figure 8 shows the estimation of the area of the hysteresis loop for all samples, which was calculated using OriginLab software. The results show that the shell-based lattice has a wider hysteresis loop, then the truss-based lattice and the plate-based lattice, respectively. Thus, the shell-based LS exhibits the greatest energy dissipation performance. This phenomenon shows that the energy dissipation of a hysteresis loop increases with the growth of the displacement as the PLA samples were compressed to a displacement level higher than that of the ABS samples, as illustrated in Figure 8.

In the end, a table is formulated comparing the specific elastic properties of the proposed architecture with those available in the literature, as shown in Table 12. There is abundant of studies available but here we mainly selected few architectures having cubic symmetry and made from polymeric materials such as ABS, PA and PLA using material extruding process (MEX). Table 12 shows that the specific Young’s modulus of the PLA/Plate-based lattice have properties like the ones obtained from PA2200/Sheet-based IWP TPMS structures. However, as per the considered cellular materials shown in table below the sheet based Neovius TPMS structures has the highest specific Young’s modulus. There is no experimentl data available in the literature that investigate the viscoelastic behavior of cellular materials with cubic symmetry, though few studies are available that characterize the time dependent response of bulk material made of PA2200 using Selective Laser Sintering technology (SLS) [55]. The authors are actively working in this area and more studies are ongoing related to the time dependent response of cellular materials.

## 4. Conclusions

In this study, the effect of the architected structural member’s geometry on the viscoelastic behavior of lattice structures with simple cubic crystal symmetry was investigated. The structural members of simple cubic LS were designed with three architectures: plate-based LS, truss-based LS, and shell-based LS. Three-dimensional (3D) printing based on material extrusion (MEX) process technology was utilized to fabricate the considered designs. The behavior of LSs were investigated for two different materials, namely, PLA and ABS. The LSs mechanical response was obtained under quasi-static compression, stress-relaxation, creep, and compressive cyclic loading tests. The obtained data was analyzed and the following conclusions are summarized:

1. From the quasi-static compression test, it was found that the plate-based LS has the greatest stiffness and strength. The shell-based LS has excellent extension but least strength. Moderate properties are observed in the truss-based lattice with a rapid fracture mechanism. In terms of materials, PLA showed greater stiffness and strength than ABS, which is due to its higher density. However, ABS showed better viscoelastic behavior at the same infill density.
2. The shell-based has the greatest normalized stress and strain over time, which indicates its remarkable viscoelastic behavior, followed by truss-based lattice then plate-based lattice. In addition, the results of compressive cyclic loading testing showed that the shell-based lattice had formed a wide load-displacement hysteresis curves, meaning it has the greatest damping performance, and energy dissipation capacity. Whereas truss-based ranked in the second, followed by the plate-based LS. By comparing the ABS and PLA materials, the better viscoelastic behavior belongs to ABS, due to its elongation and flexibility.
3. A wide variety of material properties can be achieved by controlling the design of cellular solids. A material with maximum stiffness, as demonstrated in the plate-based lattice, is valuable as an engineering material for stiffness-dominated applications and lightweight structures. Whereas a material with excellent energy dissipation response, as observed in the shell-based lattice, is a great choice to be utilized where an application requires to be designed with bending-dominated behavior.
4. This study provides the comparison of viscoelastic behavior of simple cubic LSs made of different structural members. This research methodology will open up new research paths where the researchers can explore the effect of different types of symmetries on the isotropic and anisotropic viscoelastic properties of LSs.

## Figures and Tables

**Table 1:** Specifications of PLA and ABS filaments.

Materials	Thickness	Density	Young’s Modulus	Strain at Break	Melting Temperature	Printing Temperature	Brand
ABS	1.75 mm	1.03 g/cm^3^	2 GPa	9%	245 °C	220–270 °C	RS Pro
PLA	1.75 mm	1.25 g/cm^3^	2.7 GPa	2%	150 °C	190–220 °C	Raise3D

**Table 2:** Parameters of 3D printing.

Materials	Printing Temperature	Heated Bed Temperature	Printing Speed	Extrusion Width	Infill Topology
ABS	250 °C	100 °C	50 mm/s	0.4 mm	Lines
PLA	205 °C	60 °C	50 mm/s	0.4 mm	Lines

**Table 3:** Details of considered designs.

Type	Unit Cell	Thickness	Infill Density	Lattice Structure	PLA Sample	ABS Sample
Shell-based lattice	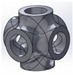	0.5 mm	27%	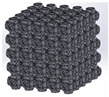	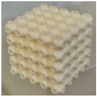	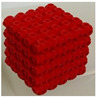
Truss-based lattice	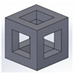	1.1 mm	27%	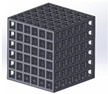	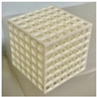	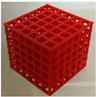
Plate-based lattice	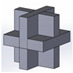	0.5 mm	27%	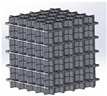	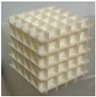	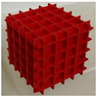

**Table 4:** ID codes of the 3D printed specimens.

Material	Geometry	Code
ABS	Simple cubic Plate-based lattice	ABS/Plate-based
ABS	Simple cubic Truss-based lattice	ABS/Truss-based
ABS	Simple cubic Shell-based lattice	ABS/Shell-based
PLA	Simple cubic Plate-based lattice	PLA/Plate-based
PLA	Simple cubic Truss-based lattice	PLA/Truss-based
PLA	Simple cubic Shell-based lattice	PLA/Shell-based

**Table 5:** Parameters of stress relaxation test.

Sample	Hold at (Displacement)	Time for Holding
ABS/Truss-based lattice	0.375 mm	30 min
ABS/Plate-based lattice	0.375 mm	30 min
ABS/Shell-based lattice	0.375 mm	30 min
PLA/Truss-based lattice	0.625 mm	30 min
PLA/Plate-based lattice	0.625 mm	30 min
PLA/Shell-based lattice	0.625 mm	30 min

**Table 6:** Parameters of creep test.

Sample	Hold at (Load)	Time for Holding
ABS/Truss-based lattice	600 N	30 min
ABS/Plate-based lattice	600 N	30 min
ABS/Shell-based lattice	600 N	30 min
PLA/Truss-based lattice	1500 N	30 min
PLA/Plate-based lattice	1500 N	30 min
PLA/Shell-based lattice	1500 N	30 min

**Table 7:** Parameters of compressive cyclic loading test.

Sample	Maximum Load	Number of Cycles
ABS/Truss-based lattice	600 N	3 Cycles
ABS/Plate-based lattice	600 N	3 Cycles
ABS/Shell-based lattice	600 N	3 Cycles
PLA/Truss-based lattice	1500 N	3 Cycles
PLA/Plate-based lattice	1500 N	3 Cycles
PLA/Shell-based lattice	1500 N	3 Cycles

**Table 8:** Weight, density, and relative density of the 3D printed specimen.

Specimen	ABS/Shell	ABS/Plate	ABS/Truss	PLA/Shell	PLA/Plate	PLA/Truss
Weight (g)	3.95	4.01	3.98	4.94	5.1	4.99
Density (g/cm^3^)	0.253	0.257	0.255	0.316	0.326	0.319
Relative density (g/cm^3^)	0.246	0.250	0.248	0.253	0.261	0.255

**Table 9:** Obtained properties from quasi-static compression test.

Specimen	Fracture Stress (MPa)	Young’s Modulus (MPa)	Specific Stiffness (MPa/(g/cm^3^))	Yield Limit Load (N)
ABS/Plate-based lattice	5.38	168	672	2563
ABS/Truss-based lattice	2.7	70	275	1481
ABS/Shell-based lattice	1.64	59.5	242	781
PLA/Plate-based lattice	12.7	443	1697	7250
PLA/Truss-based lattice	4.9	177.8	697	2750
PLA/Shell-based lattice	3.64	93.75	370	1812

**Table 10:** Obtained properties from the stress relaxation test.

Specimen	Normalized Stress (%)	Stress Relaxation Modulus (MPa)	Relative Modulus (MPa)
ABS/Plate-based lattice	17%	104	0.62
ABS/Truss-based lattice	19%	48.67	0.69
ABS/Shell-based lattice	21%	47.3	0.79
PLA/Plate-based lattice	19%	210.8	0.48
PLA/Truss-based lattice	21%	104.4	0.59
PLA/Shell-based lattice	23%	72	0.77

**Table 11:** Obtained properties from the creep test.

Specimen	Strain Increased (%)	Strain Compliance (1/MPa)
ABS/Plate-based lattice	10%	0.0086
ABS/Truss-based lattice	15%	0.0202
ABS/Shell-based lattice	19%	0.0308
PLA/Plate-based lattice	17%	0.0041
PLA/Truss-based lattice	24%	0.0095
PLA/Shell-based lattice	26%	0.0130

**Table 12:** Elastic properties comparison of cubic symmetry cellular materials.

Polymer	Architecture	E/Es	Reference:
ABS	Plate-based lattice	0.084	Current Work
	Truss-based lattice	0.035	
	Shell-based lattice	0.030	
PLA	Plate-based lattice	0.164	
	Truss-based lattice	0.066	
	Shell-based lattice	0.035	
PLA	Honeycomb-Hexagonal	0.067	Leon et al. [56]
	Honeycomb-Triangular	0.122	
ABS	Honeycomb-Trianglular	0.048	Monkova [57]
PA2200	TPMS sheet Primitive	0.082	Abueidda [58]
	TPMS sheet IWP	0.163	
	TPMS sheet Neovius	0.184	
PA1102	TPMS ligament Diamond	0.039	Abou-Ali [59]
	TPMS ligament Gyroid	0.048	
	TPMS ligament IWP	0.030	

## Data Availability

Not applicable.
